# The feasibility and reliability of actigraphy to monitor sleep in intensive care patients: an observational study

**DOI:** 10.1186/s13054-020-03447-8

**Published:** 2021-01-29

**Authors:** L. J. Delaney, E. Litton, K. L. Melehan, H.-C. C. Huang, V. Lopez, F. Van Haren

**Affiliations:** 1 School of Nursing, Queensland University of Technology, Brisbane, QLD Australia; 2 Medicine and Health Sciences, Australian National University, Canberra, ACT Australia; 3 Intensive Care Unit, Fiona Stanley Hospital, Perth, WA Australia; 4 Intensive Care Unit, St John of God Hospital Subiaco, Perth, WA Australia; 5 Sleep Unit, Royal Prince Alfred Hospital, Sydney, NSW Australia; 6 Faculty of Medicine and Health, University of Sydney, Sydney, NSW Australia; 7 Respiratory and Sleep Medicine, Canberra Hospital, Woden, ACT Australia; 8 Canberra Obesity Management Service, Canberra Health Services, Belconnen, ACT Australia; 9 School of Nursing, Hubei University of Medicine, Shiyan, China; 10 School of Nursing, University of Tasmania, Hobart, TAS Australia; 11 Intensive Care Unit, Canberra Hospital, Garran, ACT Australia

**Keywords:** Sleep, Intensive care, Polysomnography, Actigraphy, Sleep quality, Sleep disturbance

## Abstract

**Background:**

Sleep amongst intensive care patients is reduced and highly fragmented which may adversely impact on recovery. The current challenge for Intensive Care clinicians is identifying feasible and accurate assessments of sleep that can be widely implemented. The objective of this study was to investigate the feasibility and reliability of a minimally invasive sleep monitoring technique compared to the gold standard, polysomnography, for sleep monitoring.

**Methods:**

Prospective observational study employing a within subject design in adult patients admitted to an Intensive Care Unit. Sleep monitoring was undertaken amongst minimally sedated patients via concurrent polysomnography and actigraphy monitoring over a 24-h duration to assess agreement between the two methods; total sleep time and wake time.

**Results:**

We recruited 80 patients who were mechanically ventilated (24%) and non-ventilated (76%) within the intensive care unit. Sleep was found to be highly fragmented, composed of numerous sleep bouts and characterized by abnormal sleep architecture. Actigraphy was found to have a moderate level of overall agreement in identifying sleep and wake states with polysomnography (69.4%; *K* = 0.386, *p* < 0.05) in an epoch by epoch analysis, with a moderate level of sensitivity (65.5%) and specificity (76.1%). Monitoring accuracy via actigraphy was improved amongst non-ventilated patients (specificity 83.7%; sensitivity 56.7%). Actigraphy was found to have a moderate correlation with polysomnography reported total sleep time (*r* = 0.359, *p* < 0.05) and wakefulness (*r* = 0.371, *p* < 0.05). Bland–Altman plots indicated that sleep was underestimated by actigraphy, with wakeful states overestimated.

**Conclusions:**

Actigraphy was easy and safe to use, provided moderate level of agreement with polysomnography in distinguishing between sleep and wakeful states, and may be a reasonable alternative to measure sleep in intensive care patients.

*Clinical Trial Registration number* ACTRN12615000945527 (Registered 9/9/2015).

## Introduction

Sleep is an important biological function essential for survival and maintaining circadian homeostasis. Patients cared for in the intensive care are reported to experience significant sleep disturbance; characterised by sleep fragmentation and a propensity for low quality sleep comprised of stages 1 and 2 of the sleep cycle [1–4]. Polysomnography (PSG) based studies have highlighted the atypical sleep architecture of intensive care patients, and the near absence of slow wave sleep (SWS) and rapid eye movement (REM) sleep [1–7]. The etiology of sleep disturbance amongst intensive care patients is widely acknowledged and thought to be multifactorial, stemming from a combination of intrinsic and extrinsic factors. However, interventional studies have had limited success in improving the sleep architecture (the distribution of different sleep states across cycles through the night) of patients [6, 8–13].

Sleep disturbance is linked to a wide range of adverse physiological and psychological outcomes that potentially impact on patient recovery [8–14]. Emerging research suggests that sleep disturbance may be a contributing factor in the onset of delirium and prolonged mechanical ventilation, both of which have been independently attributed to increased patient mortality and intensive care length of stay [14, 15]. As a result, there is an increased awareness of the important role sleep plays in individual well-being and health, and in turn on patient outcomes.

The paradigm shift of Intensive Care Units (ICU) to be more person centred has identified sleep quality and quantity as an issue for patients receiving care, that can mitigate recovery and contribute to protracted ICU length of stay [16]. However, these person-centred aspects of care are often negated due to the rapid development, and advancement of technology that is employed within ICU intended to support survival [17]. Increasingly, the contribution of sleep in the recovery process and the overall care of the patient in the ICU, has emerged as an aspect of clinical care that has garnered considerable attention. The ability to clinically monitor sleep in the intensive care remains a challenge, secondary to issues with feasibility and accuracy of sleep monitoring methods that are commonly available [5, 18, 19]. Although PSG is considered the gold standard of sleep monitoring, its application in the intensive care setting has not been validated. Further, the interpretation of data is compounded by the abnormal electroencephalographic (EEG) activity induced by medications, and physiological changes related to critical illness [20–22]. In addition, PSG is not considered a feasible method for widespread implementation into the critical care setting due to its costs, which have been reported to be $606.35 (AUSD) per patient [23], complex set up, and the technical skills of a sleep technician required to interpret results with its application primarily limited to research.

Identifying methods to monitor and assess sleep activity is central to supporting patients physiologically and their outcomes, in addition to evaluating the effectiveness of sleep promoting interventions. Exploring the application of alternative methods for sleep monitoring is needed, in order to identify feasible methods capable of gathering accurate, and objective biophysiological data regarding sleep suitable for clinical implementation. Whilst observational assessment of sleep by nurses are considered cost effective and implementation is uncomplicated, this approach has been shown to be subject to observer bias. Studies conducted suggest that nurses overestimate the sleep that a patient acquires and are unable to identify the extent of sleep disturbance a patient may experience [24–26]. In contrast, actigraphy (ACTG) provides an objective physiological assessment via non-invasive use of motion accelerometers to detect multiplanar gross motor activity. Pre-determined algorithms translate the identified activity counts into epochs to report sleep and wakeful states [27]. This method may provide some advantages over PSG in that it is less-expensive, non-invasive and interpretation of data is less complex. In addition, it has superior data management capabilities compared with PSG, and may provide beneficial information on sleep related outcomes in response to extended interventions as it can monitor activity patterns for a period of 30 days [28].

The purpose of the study was to investigate the feasibility of 24 h continuous sleep monitoring through the application of actigraphy compared to the gold standard of sleep monitoring (polysomnography) for patients admitted to an intensive care setting.

## Methods

### Study design and setting

The study employed an observational research approach, using a with-in subject design to concurrently monitor sleep via polysomnography and actigraphy in a 31-bed tertiary referral ICU. The intensive care unit provided both medical and surgical services, inclusive of trauma, neurosurgical and cardiothoracic care, within an open plan design whereby patient care spaces are shared in either a two or four bed configuration, with a total of four isolation rooms. Patient care spaces are separated by patient curtains and semi partitioned walls separate the patient rooms. The registered nurse to patient ratio was comprised of 1:1 for intensive care patients and 1:2 for high dependency patients; at the time of the study the unit did not have in place a specific sleep promotion protocols other than offering eye masks and ear plugs for patients.

### Participants

The sample size for the study was determined using Cohens power primer previously described with an alpha value of 0.05% and a power of 80% [29]. A total of 80 patients admitted to the intensive care who met the inclusion criteria; aged 18 years or older, required an ICU admission for 24 h or greater and, whose Richard Agitation–Sedation Scale (RASS) was assessed as between  + 2 (agitated) and − 3 (moderate sedation) were recruited into the study according to previously published criteria [29]. The structured assessment of sedation and agitation via the RASS has previously been reported to have robust inter-rater reliability and validity and, is employed to assess level of consciousness and agitation amongst ICU patients [30]. Patients receiving mechanical ventilation were not excluded from the study. Rather, patients were excluded from the study based on treatment intention (palliative care), medication (admitted secondary to drug overdose, administration of neuromuscular blocking agents or barbiturates), underlying cognitive or neuromuscular degenerative disorders, and suspected encephalopathies as previously published [29]. Recruitment into the study did not impose any restrictions or amendments to the provision of clinical care, with staff being able to perform clinical care and interventions as they deemed appropriate.

Demographic and clinical data were obtained from the patient’s electronic medical record (Metavision., iMDsoft). Diagnoses at ICU admission were derived from the Acute Physiological Chronic Health Evaluation (APACHE II) classifications and the reported APACHE II score reported at admission was applied to determine the severity of illness.

### Sleep monitoring

Sleep monitoring was initiated between 13:00–16:00 h and with the aim to continue for a period of 24 h. Recording commencement between the ACTG and PSG was synchronized using the data acquisition computer.

ACTG monitoring was undertaken via Actiwatch Plus (Spectrum, Phillips Respironics), worn as a wristwatch. The device was applied to participants wrist with the least amount of invasive instrumentation (e.g. arterial line). ACTG accelerometer monitoring is based on cantilever bilayer piezoelectric sensors to detect multiplanar motor activity with forces at 0.01 g and has a sampling rate of 32 Hz. The sensitivity threshold settings for ACTG range from low (20), medium (40 and high (80) as default settings, for the purposes of this study the threshold setting applied medium. Medium is considered to recommended threshold setting based on the validation studies conducted using ACTG. Whereby, the number of activity that exceed 40 are deemed to wakeful states and 40 and under activity counts assessed as sleep.

Ambulatory PSG monitoring (Embla PSG, VMedical) commenced with skin preparation conducted according to standard techniques, and gold cup electrodes applied according to the international 10–20 electrode placement. The bedside nursing staff assigned to the patient were instructed on electrode replacement and device shut down procedure in the event of patient transfer from the ICU or due to clinical necessity (e.g. defibrillation, MRI procedure). Clinical staff were not instructed to replace electrodes in the event they became displaced. PSG data was scored by a senior sleep technologist (SST) using specialised analysis software (Remlogic), who was blinded to the results of ACTG. Further detailed information related to the sleep monitoring protocol and clinical environment is reported elsewhere [29].

### Data analysis

ACTG data was analyzed as sleep or wake at 30-s epochs via the actigraphy analysis software (Actiware version 6.0.9; Phillips Respironics), using the predetermined activity count for each epoch within the software algorithm. PSG data was downloaded using data acquisition software (Remlogic-E, version 3.4.1; Embla systems) and scored by a board-certified SST in 30-s epochs according to published criteria [31]. The SST was blinded to the results of actigraphy to avoid ascertainment bias.

The contributing factors leading to missing data from the sleep monitoring methods (PSG and ACTG) were documented and included into the analysis as a factor addressing the clinical feasibility and acceptability of the monitoring techniques. In the cases where a recording method failed, data acquired of less than 2 h were excluded from the analysis. Incomplete data was integrated in the epoch by epoch analysis of PSG and ACTG accuracy using the binary assessments of sleep or wakeful states. PSG and ACTG data were analyzed based on concurrent recording to allow for direct comparison, where data could not be directly compared this data was excluded from analysis.

Descriptive analysis reporting means and medians were applied to describe continuous data, and percentages to report categorical data. Agreement between the two sleep monitoring techniques was determined via the Kappa Cohen Coefficient to identify if the two assessment methods were reliable and not occurring secondary to chance. The outcome measure of interest in the study was to determine the accuracy of ACTG compared to PSG in reporting total sleep time, wake time, sleep percetage and extent of sleep disturbance. Sensitivity of actigraphy data was evaluated and defined as the percentages of epochs score in agreement with PSG for sleep [5]. Specificity was determined to be the percentage of agreement between actigraphy and polysomnography for epochs scored as a wakeful state [5].

Pearson Correlation coefficient was used to identify if the two measures correlated regarding the assessment of sleep and wakeful states. Bland–Altman concordance technique was applied to determine if a meaningful agreement occurred between ACTG and PSG assessments of sleep and wakeful states. Comparisons of the mean PSG and ATCG were plotted on the *x-axis*, with the difference between ACTG and PSG plotted on the *y*-axis. Statistical analysis of the acquired data was conducted via Statistical Packages for Social Sciences (Version 21), with *p* values < 0.05 deemed statistically significant.

### Ethics

Consent for participation in the study was sought either verbally or in writing from those patients who were able to provide their own informed consent. Patients who were unable to provide their own consent due to their clinical acuity, consent for participation was sought from the assigned decision-maker documented for the patient. Patients and proxy consent providers were able to withdraw their consent from the study at any point without prejudice. The study was approved by the institutions Human Research Ethics Committee (ETH.5.16.071) and was formally registered as a clinical trial with the Australian New Zealand Clinical Trial Registration Number; ACTRN12615000945527 (Registered 9/09/2015).

## Results

### Demographic profile; application and tolerability of sleep monitoring

**Fig. 1:**
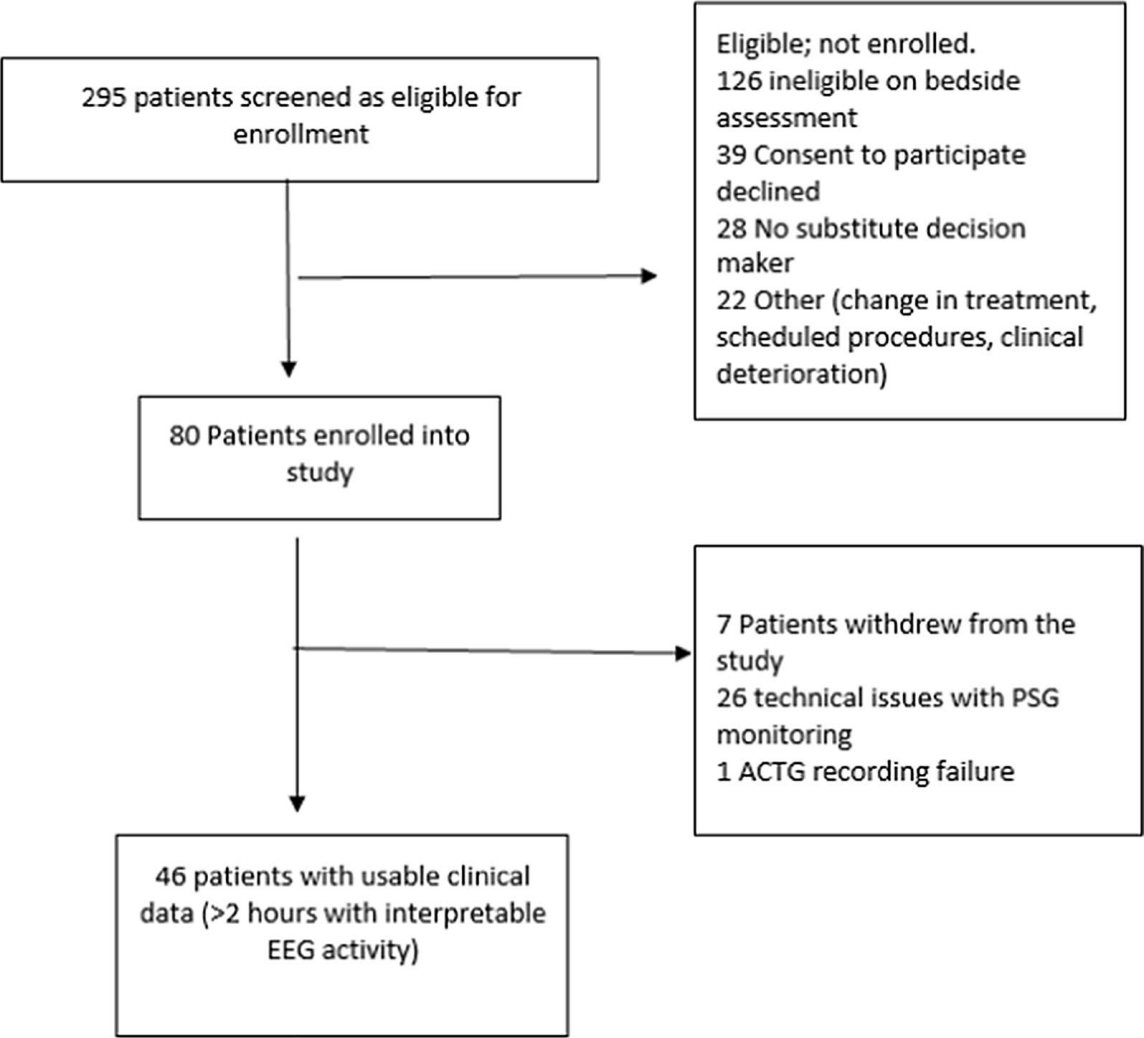
Consort diagram

A total of 295 patients were screened and 80 patients were enrolled into the study (Fig. 1). Technical issues associated with PSG monitoring such as electrode displacement (19%, *n* = 15), data transfer and equipment failure contributed to several lost studies (14%, *n* = 11). The primary reasons for early cessation of PSG monitoring once enrolled into the study was identified as patient’s inability to tolerate the monitoring technique for the 24-h duration (24%, *n* = 11). In these instances, PSG data that was obtained was used for epoch analysis between the two monitoring methods. Comparatively, ACTG monitoring was found to be less burdensome for patients and better tolerated with all participants able to maintain the ACTG monitoring for 24 h. One ACTG recording was lost due to technical issues, with all other studies having data available with intermittent loss of 1.8% of monitoring time occurring in 8 cases, attributed to removing the monitoring device to attend to patient hygiene.

The final data analysis of participants (*n* = 46) included medical and post-operative admissions with a median age of 60.5 years (IQR 45.75–72), median APACHE II score of 20.5 (IQR 14–25.25) (Table 1). Participants were primarily classified as intensive care admissions (56.5%), with 30.4% receiving mechanical ventilation during the study, and opioids were prescribed to 52% of patients.

**Table 1:** Baseline characteristics of patients

Demographic profile (*n* = 46)	
Age (years) (Median: IQR)	60.5 (45.75–72)
Gender: male/female (%, *n*)	69.6/30.4 (32/14)
Patients with known sleep disorder (%, *n*)	28.3 (13)
Weight (kg) (Median: IQR)	89 (74.75–97.25)
Height (cm) (Median: IQR)	175.5 (170–181)
BMI (kg/m^2^) (Median: IQR)	28.1 (23.83–30.9)
Clinical acuity (Median: IQR)	
APACHEII score	20.5 (14–25.75)
Relative mortality risk	36.8 (18.65–55.97)
SOFA score	13 (9–16)
RASS score (%, *n*)	
Lightly sedated (− 2)	0
Drowsy (− 1)	26.1 (12)
Calm and co-operative (0)	50 (23)
Restless (+ 1)	17.4 (8)
Agitated (+ 2)	4.3 (2)
Very agitated (+ 3)	2.2 (1)
Confusion assessment method: positive (%, *n*)	2.2 (1)
Admission classification (%, *n*)	
ICU/HDU	56.5/43.5 (26/20)
Reason for admission (%, *n*)	
Bacterial pneumonia	13 (6)
Cardiovascular	19.6 (9)
Respiratory failure	15.2(7)
Multi-trauma	10.9 (5)
Sepsis	15.2 (7)
Cardiac arrest	4.3 (2)
General surgical	13 (6)
Other	8.8 (4)
Respiratory support (%, *n*)	
Nil	8.7 (4)
Oxygen therapy	23.9 (11)
Hi Flow Nasal Prongs	32.6 (15)
Non-invasive ventilation	4.3 (2)
Mechanical ventilation	30.4 (14)
CRRT (%, *n*)	15.2 (7)
IABP	6.5 (3)
Pharmacological agents (%, *n*)	
Benzodiazepine	10.9 (5)
Opioids	52.2 (24)
Melatonin	10.9 (5)
Length of ICU stay: hours (Median: IQR)	152.5 (75–330.25)

*BMI* body mass index, *APACHEII* acute physiologic assessment and chronic health evaluation 2, *SOFA* sequential organ failure assessment, *CRRT* continuous renal replacement therapy, *IABP* intra aortic balloon pump

### Sleep characteristics

Total sleep time (TST) acquired by intensive care patients (*n* = 46) was found to be 462.5 ± 341.4 min (*M* ± SD) reported by PSG (Table 2). Sleep bouts were found to traverse both day and night over the monitoring period, with ACTG identifying an average of 15 sleep bouts. The percentage of recorded time asleep reported via PSG was 42% ± 27.3% (*M* ± SD), and was characterized by frequent awakenings (43.3 ± 35.3 per hour; *M* ± SD). Slow wave sleep and REM sleep were reduced, while the sleep–wake transition (Stage 1) was found to be increased, along with Stage N2; 75.5% of TST.

**Table 2:** Reported sleep parameters determined by polysomnography and actigraphy

Parameter	Polysomnography (*n* = 46)	Actigraphy (*n* = 46)
Total minutes analysed	37,938	37,938
Total sleep time: min (mean ± SD)	462.5 ± 341.3	508.8 ± 321.8
Wake time: min (mean ± SD)	308.2 ± 259.1	292 ± 234
Sleep fragmentation index (mean ± SD)		46.5 ± 19.7
% recording time asleep (mean ± SD)	41.8 ± 27.15	69.2 ± 19.4
Awakenings (mean ± SD)	43.3 ± 35.3	19.8 ± 19.3
Arousal index	14.3 ± 13.1	
Stage N1 (%)	10.4	
Stage N2 (%)	75.5	
SWS (%)	10	
REM (%)	4.1	

*SWS* slow wave sleep, *REM* rapid eye movement

### Actigraphy monitoring of sleep

There was a moderate level of agreement between PSG and ACTG independent assessment of sleep and wakeful states (69.4%). Adjusting for the probability of chance, the agreement between to the two assessments was found to be statistically significant (*ĸ* = 0.386, *p* < 0.05), identifying a moderate level of correlation between accuracy of ACTG compared with PSG for assessing sleep and wake states (*r* = 0.368, *p* < 0.00). A moderate level of specificity (agreement in the identification of wakefulness) was identified between PSG and ACTG (76.1% agreement), and sensitivity for the agreement between the assessments for sleep (65.5%). Sensitivity and specificity of actigraphy was found to fluctuate depending on ventilation status and the time-period being monitored (daytime 06:00–22:00 h compare to night-time 22:00–06:00 h), with non-ventilated patients having higher percentages of specificity and sensitivity (Table 3).The administration of benzodiazepines and opioids amongst ventilated and non-ventilated patient were not identified as a significant fact or between the groups (*Ӽ*^2^ = 0.351 and *Ӽ*^2^ = 0.148 respectively). Rather a higher APACHEII score (> 20) was linked to mechanical ventilation (*p* > 0.05).

**Table 3:** Specificity and sensitivity of actigrpahy based on mechanical ventilation requirements

	Specificity (%)	Sensitivity (%)	Kappa Cohen
Study cohort	76.10	65.50	0.386 *p* < 0.05
Patient receiving mechanical ventilation	51	84.10	0.363 *p* < 0.05
Non- mechanically ventilate patients	83.70	56.70	0.371 *p* < 0.05
Daytime hours (06:00–22:00)	84.10	54.60	
Night-time hours (22:00–06:00)	52.90	78.30	
Daytime hours (06:00–22:00) + non-ventilated patients	90.20	41.40	
Night-time hours (22:00–06:00) + non-ventilated patients	62.30	73.50	
Daytime hours (06:00–22:00) + mechanical ventilation	60.90	80.00	
Night-time hours (22:00–06:00) + mechanical ventilation	32.30	89.70	

Concurrent ACTG analysis of sleep and wake states compared to PSG was found to correlate the reported total seep times (*r* = 0.873, *p* < 0.01) with ACTG over reporting total sleep time, and wakefulness (*r* = 0.769, *p* < 0.01) over reported by ACTG (Table 4). Post hoc analysis of mechanically ventilated patients compared to non-mechanically ventilated patients identified a reduction in ACTGs ability to distinguish wakefulness [*r* = 0.565, *p* < 0.05: non-mechanically ventilated (*r* = 0.788, *p* < 0.00)]. Whilst, TST reported by PSG and ACTG was found to have a high correlation amongst both mechanically ventilated patients (*r* = 0.924, *p* < 0.000), and non-mechanically ventilated patients (*r* = 0.841, *p* < 0.00). Sleep percentage retained a low level of correlation between PSG and ACTG in non-mechanically ventilated patients (*r* = 0.411, *p* < 0.05).

**Table 4:** Correlations of Polysomnography and Actigraphy sleep parameters

	ACTG wake time	ACTG TST	ACTG sleep percentage	ACTG awakening	ACTG fragmentation index
PSG Wake time	0.769**	0.052	0.045	0.408**	0.338*
PSG TST	− 0.079	0.873**	0.375*	0.323*	− 0.262
PSG sleep percentage	− 0.233	0.687**	0.411*	0.269	− 0.359*
PSG awakening	0.298*	0.308*	0.139	0.227*	0.052
PSG arousal index	0.257	− 0.204	− 0.051	0.101	0.322*

**p < 0.00; *p < 0.05

**Fig. 2:**
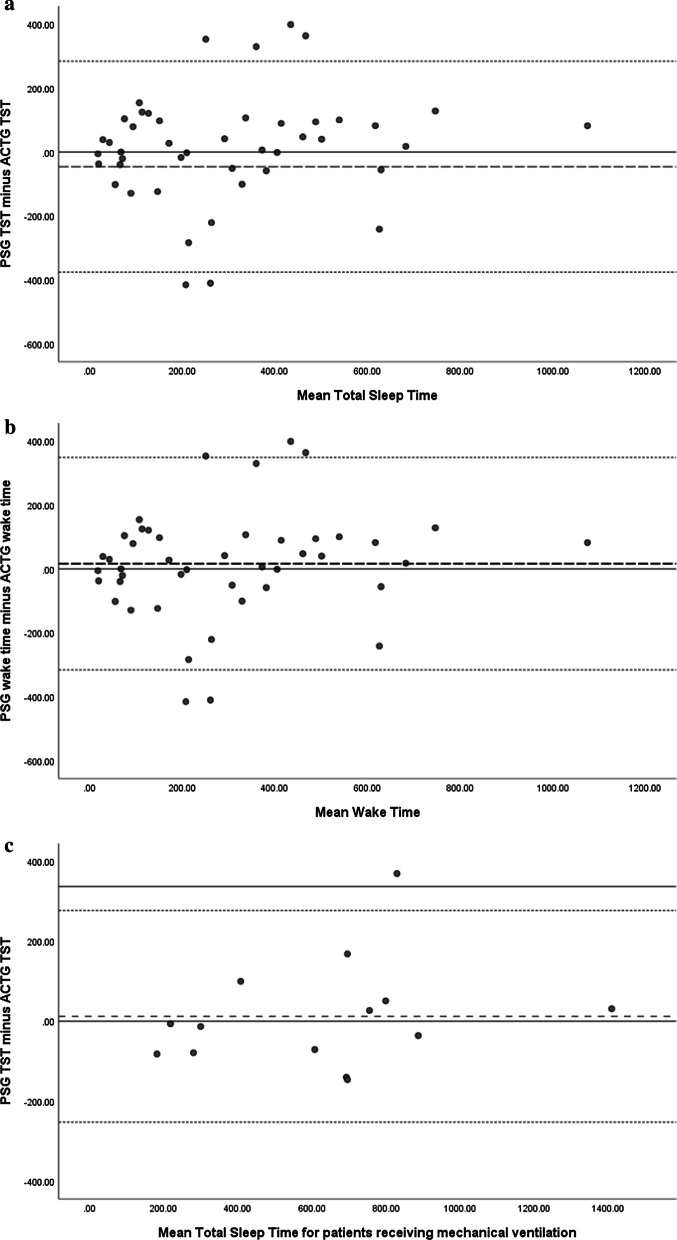

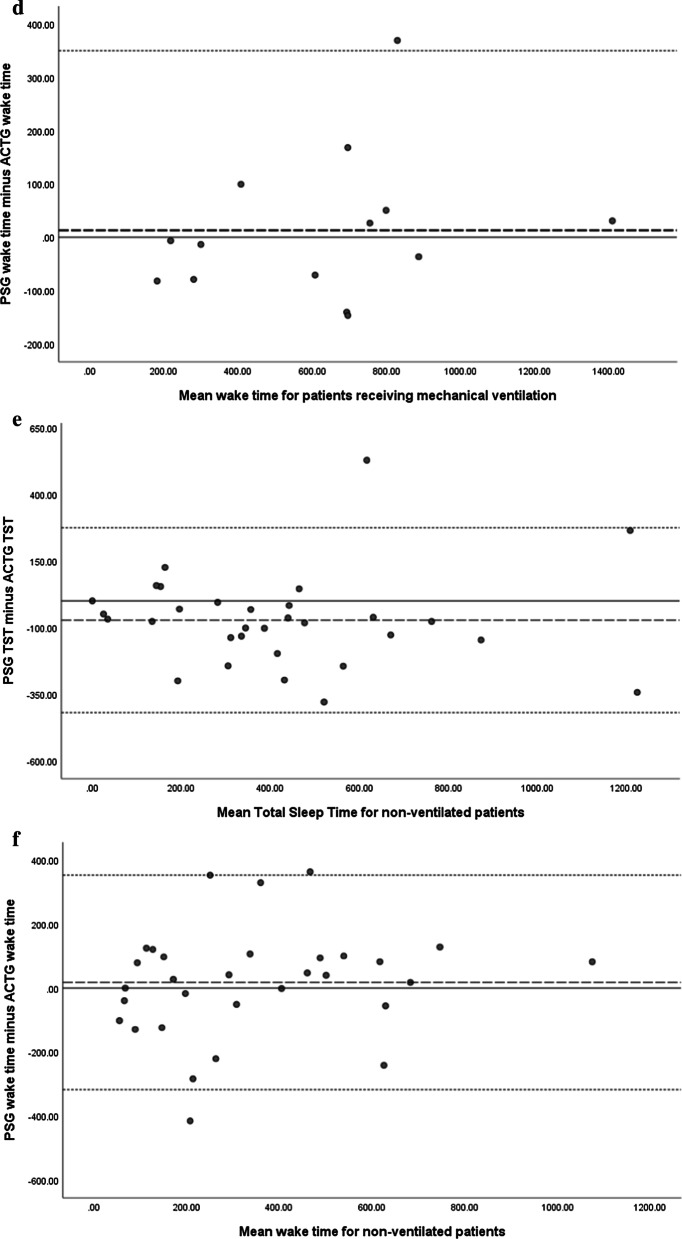
Bland–Altman plots: Horizontal line drawn at the mean difference and at the mean difference plus and minus 1.96 times the standard deviation of the differences, with the y-axis reporting mean duration determine by polysomnography. **a** Polysomnography versus Actigraphy assessment of total sleep time. **b** Polysomnography versus Actigraphy assessment of wakeful states for the study cohort. Graphs **c** and **d** depict Polysomnography versus Actigraphy assessment of total sleep time amongst mechanically ventilated patients (**c**) and Polysomnography versus Actigraphy assessment of wakeful states for patients receiving mechanical ventilation (**d**). Non-ventilated patient total sleep time (PSG vs ACTG), and wake time (PSG vs ACTG) is represented in graph **e** and **f**

Bland–Altman plots of total sleep time and wake time indicated that ACTG overall overestimated TST by 46 min and underestimated wakeful states (16 min) (Fig. 2). The overestimation of TST was further increased amongst non -ventilated a patient (73 min) and remained underestimated for wakeful states (18 min). The large standard deviation identified with the data is reflected by the level of agreement and suggest that there is large variability between the two measures. Linear regression analysis was performed on the assessments of wake time and total sleep time assessed by polysomnography compared to actigraphy amongst the group analysis indicated an absence of proportional bias (*p* > 0.05).

## Discussion

Identifying a feasible objective physiological assessment of sleep is necessary to understand the impact of sleep disturbance on intensive care patients. This study undertook a comparison of sleep monitoring techniques to assess sleep and wakeful states using the accepted gold standard of sleep monitoring; polysomnography and motion accelerometer monitoring via actigraphy. Findings suggest that ACTG has a moderate level of sensitivity and specificity in identifying binary states of sleep and wakefulness, compared to PSG. Ventilation status (mechanical ventilation: non-mechanically ventilated) of patients was found to impact on the accuracy of the ACTG in distinguishing sleep and wake states. However, this was not found to be attributed to the administration of opioid or benzodiazepine infusions, rather higher APACHEII scores were linked to the need for mechanical ventilation. ACTG was found to have moderate to high levels of correlation with PSG in identifying sleep duration and wakefulness in non-ventilated patients.

Sleep is increasingly recognised as an important factor in patients’ overall well-being and recovery, resulting in its integration into the Clinical Practice Guidelines for the Preventions and Management of Pain, Agitation/Sedation and Delirium developed by the Society of Critical Care Medicine [32]. Despite its inclusion, the ability for clinicians to feasibly monitor sleep and evaluate the effectiveness of sleep protocols and interventions has been problematic. However, our findings suggest ACTG may provide a surrogate method that can be clinically applicable amongst select ICU patients. This study is one of the first studies to report the feasibility of ACTG as a sleep monitoring technique within ICU, to assess sleep duration and disruption compared to PSG. Findings indicate that ACTG provides a clinically viable method for non-ventilated patients that is minimally invasive, easy to establish unattended monitoring and interpret findings [28].

Previous research in non-critical care contexts has validated ACTG against PSG (level of agreement > 90%) in distinguishing sleep and wake states [33, 34]. However, its application in critically ill patients is limited, with previous research conducted by Beecroft et al. [5] and van der Kooi et al. [19] reporting low levels of agreement between PSG and ACTG. The findings of this study contest these and report an overall moderate level of agreement (69.4%), and accuracy to identify sleep (65.5%) and wakeful states (76.1%). The non-ventilated patient subset were found to have a improved level of agreement, which may be attributed to lower APACHEII scores (< 20), a reduction in the administration of opioids and benzodiazepines and RASS scores within the cohort. Sensitivity and specificity were found to vary considerably based on ventilation status with an increased specificity to identify wakeful states in non-ventilated patients and an increased sensitivity towards identifying sleep in mechanically ventilated patients. ACTG accuracy appears to decline when physiological activity is reduced which is consistent with mechanically ventilated patients. The findings of this study suggest that higher APACHEII scores may be an impacting factor on the accuracy of ACTG compared to PSG.

ACTG monitoring of non-ventilated patients reported a higher level of correlation to PSG in detecting wakeful and sleep states. This may be related to this patient demographic having more distinct physiological activity consistent with increased movement during wakefulness, with distinct reductions during sleep. In contrast, ACTG was found to correlate to PSG in reporting sleep states amongst mechanically ventilated patients, with a poor ability to detect wakefulness. This patient cohort are the greatest challenge in monitoring as spontaneous movement is frequently reduced due to pharmacological agents, higher clinical acuity, and atypical EEG activity which may be present in patients with higher APACHII scores. As a result, ACTG’s inability to distinguish between sleep and diminished physiological activity during wakeful states contributes to an erroneous interpretation. Mechanically ventilated patients within this study were assessed has having prolonged total sleep times, with limited arousals and spontaneous movements detected by both PSG and ACTG. The findings suggest that there is considerable variability between the two measures with ACTG having a modest level of agreement with PSG. The clinical application of ACTG for sleep monitoring may be more appropriate for non-ventilated patients cared for in the Intensive Care, whose wake states are observable.

Actigraphy was found to overestimate sleep and underestimate wakefulness which reiterates previously published findings [5, 19, 35]. The sleep architecture of patients in this study was found to be atypical despite limited administration of benzodiazepines and less than 50% of the recruited patients requiring mechanical ventilation. Sleep was comprised of primarily stage N2 sleep and highly fragmented, with limited restorative components identified (SWS and REM sleep), which has been previously reported amongst ICU patients. Actigraphy’s overestimation of sleep has been purported to be related to its lack of sensitivity in distinguishing between sleep, motionless resting activity and movement occurring during sleep or the provision of clinical care [5]. Specifically, how the commencement of sleep is recognised may be a critical factor impacting on accuracy. With ACTG denoting immobility as the beginning phase of a sleep period, whereas PSG identifies changes in neuroelectrophysiological activity patterns marking the onset of sleep. As these changes commence well after a period of wrist immobility, actigraphy is frequently reported to overestimate sleep time. This finding has been reported to be more pronounced in those patients with abundant wakefulness through the night [36]. The presence of sedating medication often effects EEG characteristics, which limited the accuracy of distinguishing between traditional sleep stages. When this occurred stage N2 was used as a default stage to indicate sleep.

The findings of this study affirm the challenges encountered in monitoring sleep via PSG within this cohort. As sleep traverses across the 24-h spectrum maintaining electrode placement over this duration is challenging as identified in this study. Further, PSG tethers patients to additional invasive monitoring, and increases the logistical complexities of providing patient care and increases patient discomfort. Technical issues included both poor signal quality due to electrode instability, which was exacerbated by the provision of clinical care in manoeuvring patients on and off the bed, as well as data loss due to the implications of the complexity of recording PSG. These factors frequently resulted in studies with usable data of less than 14 h, and highlight that PSG is suitable for widespread clinical implementation. As a result, it is imperative that assessment methods are identified that are both feasible and capable of prolong monitoring and, imposes a limited burden to the patient and does not further increase the complexity of providing patient care is required. Recording tolerance was identified as an issue amongst conscious patients with requests to terminate monitoring upon waking from the night of sleep. This was consistent with findings reported by Knauret et al. [18] who reported 31% of medical ICU patients experience issues with monitoring tolerance related to discomfort. The combination of technical issues and patient acceptability of PSG monitoring reiterates that it is an unfavourable approach for widespread sleep monitoring in the ICU and may exacerbate sleep disturbance as a result.

Acknowledging the complexities of PSG monitoring and the limitations of ACTG in terms of patients with limited mobility and higher sedation scores are important considerations when implementing current available sleep monitoring techniques. Judicious assessment of patients should be considered when implementing ACTG for ICU sleep monitoring. In this study the devised exclusion criteria were developed based on previously reported sleep research, and the identified factors that can confound electroencephalographic activity and interpretation. The sedation level primarily reported in this study was a RASS score of zero (calm and cooperative), limited benzodiazepine administration, reduced requirement for invasive mechanical ventilation support, and lower APACHII scores may be critical factors that aided in ACTG’s ability to track patients sleep–wake patterns more accurately. As a result, ACTG monitoring was not implemented in clinical cases where it would have been deemed futile secondary to immobility and high sedation levels.

Sleep disturbance is recognised as a clinical issue that can negatively affect the rehabilitation of ICU patients and their engagement in activities aimed at expediating their recovery [35]. Furthermore, functional, and cognitive recovery has been reported to be impeded amongst patients who are subjected to sleep disturbance and fragmentation [36, 37]. The important role sleep has in functional recovery is supported by clinical research which indicates that addressing sleep disturbance amongst critically ill patients leads to reduced disability at discharge [38, 39]. Patients capable of spontaneous movement appear to be vulnerable to high levels of sleep disturbance and are potentially a target population within the ICU for sleep promoting interventions. Although, ACTG will not provide detailed information on sleep architecture, its potential application for monitoring sleep characteristics for those with protracted ICU admission may be of value, in that it can track sleep–wake cycles over extended durations, along with assessing the potential response to sleep supporting interventions.

### Limitations

The findings reported in this study need to be considered before extrapolating them more widely, acknowledging that the research involved a single study centre, and as a result the findings may not be transferrable to other Intensive Care Units who have their own unique features and patient demographics. The research site employs a minimal sedation and early mobilisation practice in the provision of care, and as a result, patients may have a lower RASS scores making patients more aware and interactive with their environment.

The implemented exclusion criteria for the study prohibited the recruitment of the most critically ill patients from participating in the study, and the findings cannot be transferred to this patient cohort. The ability to effectively monitor sleep in heavily sedated patients remains clinically challenging, with PSG likely to be the most appropriate assessment method currently available. It may be contended that in most critically ill patients within the ICU the focus of care is primarily on preserving life, and the sleep quality and quantity may not be a critical factor at this point of care. Rather, sleep may be a critical factor in the transitioning of care to recovery, weaning from mechanical ventilation and rehabilitation from critical illness whereby, sleep disturbance and deprivation may contribute to the potential onset of delirium [24, 40–43]. It is amongst this cohort, that ACTG may be most appropriate to implement. ACTG monitoring is not infallible and its limitations need to be acknowledged in terms of its potential to distinguish between voluntary and involuntary movements. Whereby, patients’ wakeful inactivity may be identified as rest or sleep, and activity associated with care interventions such as repositioning may be inaccurately identified as a wakeful state [44].

## Conclusions

Actigraphy was found to have a moderate level of agreement with PSG in distinguishing between sleep and wakeful states. There is a large variability between the two measures of sleep amongst both mechanical ventilation and non-ventilated patients. Correlations between ACTG and PSG were stronger amongst non-ventilated patients compared to those requiring mechanical ventilation. Although PSG is the gold standard for sleep monitoring its feasibility is debatable in the ICU environment when sleep traverses the 24 h. ACTG offers a modest level of agreement in identifying sleep, wakefulness, and sleep disturbance, was found to be more tolerable and less cumbersome than PSG, and data loss was minimal. Consideration should be applied to patient selection for ACTG sleep monitoring, with patients whose care has transition to recovery with reduced sedation being potential candidates. Future research is needed to ascertain the transferability of research findings to broader ICU environments that may have their own unique characteristics. Whilst ACTG is not able to provide information regarding sleep architecture, it does provide clinicians with useful objective biophysiological assessment of sleep quantity and fragmentation.

## Data Availability

The datasets analyzed during the current study are not publicly available due to the clinical study report being finalized but will be available from the corresponding author on reasonable request at a later time.

## Abbreviations

ACTG: Actigraphy
ACTRN: Australian Clinical Trials Registration Number
APACHII: Acute Physiologic Assessment and Chronic Health Evaluation 2
AUSD: Australian Dollars
BMI: Body Mass Index
CCRT: Continuous renal replacement therapy
EEG: Electroencephalogram
ETH: Ethics
HDU: High Dependency Unit
Hz: Hertz
IABP: Intra-aortic balloon pump
ICU: Intensive Care Unit
IQR: Inter quartile range
*K*: Kappa
*M*: Mean
MRI: Magnet Resonance Imaging
*N*: Number
PSG: Polysomnography
*R*: Pearson correlation
RASS: Richard Agitation–Sedation Scale
REM: Rapid eye movement
SD: Standard deviation
SOFA: Sequential organ failure assessment
SST: Senior Sleep Technologist
SWS: Slow wave sleep
TST: Total Sleep Time
